# Editorial: Hydrothermal and submarine volcanic activity: impacts on ocean chemistry and plankton dynamics

**DOI:** 10.3389/fmicb.2025.1703123

**Published:** 2025-10-09

**Authors:** Sophie Bonnet, Cecile Guieu, Ilana Berman-Frank, Douglas G. Capone, Jessica Nicole Fitzsimmons, Joseph A. Resing

**Affiliations:** ^1^Aix Marseille University, Université de Toulon, CNRS, IRD, MIO, Marseille, France; ^2^Sorbonne Université, Centre National de la Recherche Scientifique, Laboratoire d'Océanographie de Villefranche, LOV, Villefranche-sur-Mer, France; ^3^Department of Marine Biology, Leon H. Charney School of Marine Sciences, University of Haifa, Haifa, Israel; ^4^Department of Biological Sciences, University of Southern California, Los Angeles, CA, United States; ^5^Texas A&M University, College Station, TX, United States; ^6^Cooperative Institute for Climate, Ocean, and Ecosystem Studies, University of Washington, Seattle, WA, United States; ^7^NOAA-Pacific Marine Environmental Laboratory, Seattle, WA, United States

**Keywords:** hydrothermal activity, iron, plankton, trace metals, ocean

Iron (Fe) is a key limiting factor for primary productivity across vast regions of the global ocean (Tagliabue et al., 2017; Browning and Moore, 2023). Traditionally, atmospheric dust deposition and continental margin fluxes have been considered the dominant sources of Fe to surface waters (Moore et al., 2004). However, deep-sea hydrothermal systems also release substantial amounts of dissolved Fe (dFe) into the overlying water column via hydrothermal plumes. These plumes can be transported over long distances across ocean basins, thereby influencing large-scale ocean chemistry (Nishioka et al., 2013; Saito et al., 2013; Fitzsimmons et al., 2014; Resing et al., 2015). Furthermore, global biogeochemical models estimate that hydrothermal fluxes from mid-ocean ridges contribute nearly 23% of the dFe inventory in the oceanic water column (Tagliabue et al., 2017).

Unlike deep hydrothermal systems associated with mid-ocean ridges (>2,000 m), hydrothermal activity can also occur at much shallower depths, such as in proximity to island arcs and hotspot volcanoes, thereby delivering substantial fluxes of trace metals to the upper ocean waters (ca. 500–1,000 m; Massoth et al., 2007; Hawkes et al., 2014) and in some cases directly to the euphotic zone (0–150 m; Chemine et al., 1991; Resing et al., 2009; Santana-Casiano et al., 2016; Guieu et al., 2018; Tilliette et al., 2022). However, unlike deep hydrothermal plumes, dFe concentrations in these shallow environments decrease rapidly with distance from the source due to the much stronger water mass dynamics that prevail at shallow depths (Tilliette et al., 2022). In deep hydrothermal systems, particulate dFe losses are mitigated through stabilization with organic Fe-binding ligands (Bennett et al., 2008; Toner et al., 2009). In contrast, in shallow hydrothermal systems, both the concentration and chemical nature of these ligands are poorly characterized, thereby constraining our ability to assess Fe stabilization, residence times, and bioavailability in these environments.

This Research Topic includes, among other contributions, several studies investigating the occurrence, composition, and functional role of ligands in stabilizing Fe near the shallow hydrothermal vents of the Tonga-Kermadec arc (Western subtropical South Pacific). Mahieu et al. identified elevated conditional concentrations of Fe-binding ligands peaking near hydrothermal sites, primarily composed of intermediate-strength L2 types. Despite this, their analysis revealed that ligand concentrations were largely in excess relative to DFe, suggesting limited effectiveness in stabilizing hydrothermal Fe inputs. Consistent with this finding, Portlock et al. reported unusually high concentrations of reduced sulfur substances (phytoplankton-derived biomolecules that associate with trace metals under elevated exposure levels), thereby mitigating toxicity. Complementing these studies, Dulaquais et al. investigated the contribution of soluble humic-like substances produced during phytoplankton degradation to Fe complexation at various sites impacted differently by hydrothermal fluids. The authors showed that the humic-ligands were unsaturated likely due to their inability to access colloidal DFe, Fe(II) and FeOx species. The humic ligands only complexed 1–5.5% of the total DFe pool close to the vent, thereby solubilizing only a small fraction of the hydrothermal Fe released. Beyond Fe, hydrothermal vents release a variety of trace elements and gases. Zhao et al. investigated the impact of hydrothermal activity on the barium cycle. They showed that Ba isotopes in vent waters and sediments are lighter than those in the water column, indicating the preferential removal of lighter isotopes during fluid–seawater mixing and highlighting their value as tracers of hydrothermal influence on sediments. In a shallow Southern Ocean bay, Belyaev et al. demonstrated how hydrothermal activity alters seawater biogeochemistry by linking vent inputs with elevated concentrations of vanadium, cobalt, nickel and Fe, along with methane and carbon dioxide. In addition to the continuous hydrothermal inputs, sporadic volcanic eruptions in many of these systems also deliver material and chemical elements. Chavagnac et al. showed that strontium (Sr) and lithium (Li) isotopic signatures can distinguish between volcanic and hydrothermal sources. Volcanic activity boosts the total Sr and Li through ash dissolution, while hydrothermal inputs drive concurrent increases of both elements in the water column.

These studies suggest that, although shallow hydrothermal systems provide substantial amounts of Fe and other trace elements, limited ligand stabilization may restrict their persistence in surface waters. This, in turn, raises important questions about the residence time, bioavailability, and ecological impact of hydrothermal Fe on plankton communities.

How do these shallow hydrothermal environments (< 500 m deep) impact phytoplankton dynamics? The proximity of Fe release to the euphotic layer greatly increases the likelihood of a direct and immediate biological response. Evidence from the Tonga–Kermadec arc indicates that Fe-rich fluids entering surface waters can stimulate intense diazotrophic activity, thereby fueling enhanced net community production, N_2_ fixation, and ultimately increased carbon export to the deep ocean (Bonnet et al., 2023). Strikingly, the carbon sequestration efficiency observed in these natural, shallow-vent systems is higher than that of artificial, mesoscale Fe-fertilization experiments, underscoring the ecological significance of shallow hydrothermal inputs as natural “fertilization hotspots” for surface ecosystems.

This Research Topic brings together several complementary studies that explore the complex biological responses elicited by the diverse suite of chemical elements supplied through hydrothermal inputs. Tilliette et al. demonstrated experimentally that increasing fluid inputs initially exerts toxic effects on planktonic communities, but subsequently stimulates net community production, N_2_ fixation, and enhanced export relative to controls. Consistent with the findings of Portlock et al., this fertilization effect is likely sustained by planktonic detoxification via thiol-based ligands that bind toxic trace metals (e.g., Cu, Cd, and Hg). Mériguet et al. provided *in situ* evidence that these shallow hydrothermal inputs structure the entire ecosystem's trophic dynamics. Using imaging and acoustic approaches, the authors demonstrated that the elevated diazotrophic biomass stimulated by hydrothermal fluids propagates through meso- and macrozooplankton communities, which in turn may enhance organic matter export via the production of fast-sinking fecal pellets. Consistent with this, Ababou et al. showed that zooplankton-derived material (fecal aggregates, carcasses, and cylindrical fecal pellets) represents more than 90% of the carbon flux exported below the euphotic zone in the vicinity of hydrothermal vents. In parallel, nitrogen isotope budgets analyzed by Forrer et al. revealed that this export is predominantly fueled by diazotrophy, which requires high Fe availability.

Together, these findings indicate that, in this system, newly fixed nitrogen is efficiently transferred up to zooplankton and repackaged into dense fecal pellets. This highlights an overlooked but highly effective pathway by which diazotroph activity indirectly sustains particulate organic carbon export, strengthening the coupling between nitrogen fixation and the efficiency of the biological carbon pump in shallow hydrothermal regions. In the much deeper environments of the Arctic Ocean, Wegener et al. reported a clear biological response within hydrothermal plumes, characterized by elevated carbon fixation rates compared to surrounding waters, suggesting enhanced chemoautotrophy fueled by hydrogen and sulfide as energy sources.

Taken together, the studies reported in this Research Topic reveal that the fate of hydrothermal Fe in shallow systems is governed by a delicate balance between its limited chemical stabilization by organic ligands and rapid biological utilization in the euphotic zone. While the weak role of binding ligands may constrain the long-term persistence of hydrothermal Fe, its immediate availability fuels primary productivity, diazotroph activity and production, trophic transfer, and greater carbon export. This coupling of trace metal chemistry and biological responses underscores the role of shallow hydrothermal systems as natural laboratories where geochemical processes and ecosystem functioning are tightly interconnected, with potentially significant implications for the oceanic carbon and nitrogen cycles.
